# The Plea of Insanity in Criminal Cases
*Remarks on the Plea of Insanity, and on the Management of Criminal Lunatics. By Wm. Wood, M.D., Medical Officer of Bethlem Hospital.On the Classification and Management of Criminal Lunatics. By John Charles Bucknill, M.B. London, Superintendent of the Devon County Lunatic Asylum.


**Published:** 1852-01-01

**Authors:** 


### Art. VI.—THE PLEA OF INSANITY IN CRIMINAL CASES.*

Is there any legal test of insanity, defined by statute, or established by precedent, to guide the course of criminal justice in its dealings with every accused person alleged to be insane? To some this question may appear captious—to others, foolish; nevertheless, we put it. Has the criminal law of this country any established test of the state of mind of every accused party with reference to the crime committed? If it has, we humbly confess our ignorance of it, and admit the impertinence of our question.

Probably we shall be told that it has; that Lord Erskine established *delusion* to be the true characteristic of insanity, and that since his time delusion has been received by the courts as the test of that condition. This is positive so far as it goes; but does it fully answer the question? Is delusion so invariably a symptom of insanity that the disorder cannot be said to exist without it? Such, indeed, was Lord Erskine's opinion. He laid it down that there could be no insanity without delusion; he said, that the person, who enjoying perfect use of his senses, interprets the evidence of his senses in the common manner, has no delusion, and cannot, therefore, be insane. And Sir John Nicholl elucidated this view very clearly in the well-known case of "Dew *v.* Clarke;" he said—

"The true criterion, the true test of the absence or presence of insanity, I take to be the absence or presence of what, used in a certain sense of it, is compressible in a single term—namely, *delusion*." . . . . "In the absence of anything in the nature of delusion, the supposed lunatic is, in my judgment, not properly or essentially insane."

The same opinion was also quoted approvingly by Lord Brougham, in the Gibson will case; and Lord Denman, in his charge to the jury in "Regina *v.* Smith," for murder, observed that—

"To say a man was irresponsible, without positive proof of any act to show that he was labouring under some *delusion*, seemed to him to be a presumption of knowledge which none but the great Creator himself could possess."

Moreover, Sir John Nicholl laid it down that mere eccentricity, or great caprice, or violence of temper, is not enough to constitute mental unsoundness; but there must be a delusion, which he defines to be "a belief of facts which no rational person would have believed." This

* Remarks on the Plea of Insanity, and on the Management of Criminal Lunatics. By Wm. Wood, M.D., Medical Officer of Bethlem Hospital.
On the Classification and Management of Criminal Lunatics. By John Charles Bucknill, M.B. London, Superintendent of the Devon County Lunatic Asylum.

definition, as amended by Lord Brougham, and expressed to be "a belief of things as realities, which exist only in the imagination of the patient," may be accepted as the legal meaning of the term delusion. So far all is clear; an insane person is a person subject to a delusion, and we are told what a delusion is. But have the courts always observed this rule, and invariably refused to admit the plea of insanity unless a delusion could be proved? We find it has not been so. Although, on the one hand, in the case of Smith, convicted and executed for the unpremeditated murder of a woman, whom he met accidentally, and who was perfectly unknown to him, we have the plea of insanity indignantly rejected by Lord Denman, on the express ground that no delusion had been proved; yet, on the other hand, in the case of Ovenston, tried before Justice Maule for the premeditated murder of a man with whom he had quarrelled, and against whom he bore malice, the prisoner was acquitted on the ground of insanity, although no distinct delusion was proved. We are firmly persuaded of the justness of the verdict in the latter case; and it would be easy to cite many equally just decisions in which the plea of insanity has been admitted, although no delusion had been shown.

Furthermore, in the trial of the case "Bainbrigge *v.* Bainbrigge," Lord Campbell distinctly said, "There may be mania without delusion." Presuming that his lordship employed the term mania in its general sense, as synonymous with insanity, we would ask what proof does the law admit of this kind of insanity? For having been told that delusion is the only recognised legal test of insanity, we are anxious to know how a case of insanity, in which no delusion exists, can be proved.

Medical psychologists, since the time that Pinel wrote of *manie sans délire* have been well aware that "mania may exist uncomplicated with mental delusion" (Hoffbauer), and equally well aware that lawyers have commonly refused to listen to their evidence on this point. We are well pleased, therefore, to find Lord Campbell saying, in July, 1850, "there may be mania without delusion," notwithstanding that Lord Denman, in March, 1849, had stated such an opinion "to be a presumption of knowledge which none but the great Creator himself could possess." The expounders of the law are sometimes pleased to be jocose on the difference of opinion among medical men, and no later than last month, Lord Chancellor Truro deliberately asserted that medical witnesses invariably gave evidence in favour of the party which pays them; but we have adduced proof that difference of opinion is not confined exclusively to "those doctors;" and we, in our turn, might make merry did it not happen that the difference of opinion among chief-justices, sometimes involves the hanging of a man, and, therefore, is no joking matter. Returning to our question, we again ask what is the legal test of insanity unaccompanied by delusion? We believe there is no test; consequently, as there are very many cases of insanity without delusion, we repeat our assertion, that, practically speaking, the law has no test, *universally* applicable, by which to try the alleged insanity of an accused party. Dr. Bucknill, in his pamphlet, mentions that,

Lord Campbell, in a debate in the House of Lords, after alluding to his "very long and very large attention to the subject," said, "he had looked into all the cases that had occurred since Arnold's trial, 1723, and to the directions of the judges in the case of Lord Ferrers, Bellingham, Oxford, Francis, and M'Naughten, and he must be allowed to say that there was a wide difference, both in meaning and in words, in their description of the law."—*Hansard's Parliamentary Debates*, vol. lxvii., p. 92.

We have pointed out one of the differences to which Lord Campbell alludes; we proceed to point out another. At the trial of Hadfield, the presiding judge, Lord Kenyon, expressed this opinion: "If a man be in a deranged state of mind at the time of committing an act, he is not criminally answerable; the material part of the case is, whether, at the very time his mind was sane,"—*i.e.*, according to the legal definition of sanity,—"his mind was free from any delusion. Now, supposing that the accused is clearly proved to have laboured under some delusion at the time he committed the crime, consequently was legally insane; nay, more, suppose that it is shown 'that the act in question was the immediate unqualified offspring of the disease' (Erskine), does that exempt the accused from legal responsibility? Lord Erskine, and the majority of his contemporaries, thought it did; not so, however, our modern judges; in their answers to the questions proposed to them by the House of Lords, relative to the case of M'Naughten, they stated: 'The opinion of the judges was, that notwithstanding the party committed a wrong act while labouring under the idea he was redressing *a supposed grievance or injury*, or under *the impression* of obtaining some public or private benefit, *he was liable* to punishment.' And in answer to the question 'If a person under an insane delusion as to existing facts, commits an offence in consequence thereof, is he hereby excused?' also said, 'If the delusion *were only partial*, the party accused *was equally liable* with a person of sane mind. If the accused killed another in self-defence, he would be entitled to an acquittal; but if the crime were committed for any *supposed injury, he would then be liable* to the punishment awarded by the laws to his crime.' By this rule, if a man subject to a single delusion, to wit, that such a one had debauched his wife, actuated by his delusion, were to kill that person in revenge for '*the supposed injury*' he would, nevertheless, be liable to punishment, notwithstanding he had been proved insane, and his act shown to be the 'unqualified offspring of his disease.' And if in such a case the friends of the prisoner should set up the plea of insanity, and prove the previous existence of the delusion, they would only aggravate his position by establishing malice prepense."

Fortunately for humanity, the practice of the law does not always accord with the theory, nor do juries invariably follow the direction of the bench; and we very much doubt if twelve men could be found to convict a man of a crime which had been shown to be the "unqualified offspring" of an insane delusion.

It was, perhaps, a perception of the practical inefficacy of the law to reach that class of insane offenders which it does not exempt from punishment, which led our judges to require proof of something more than the mere existence of insanity. For they decided—and never was a legal opinion more carefully considered, or more deliberately pronounced—"that before a plea of insanity should be allowed, undoubted evidence ought to be adduced that the accused was of *diseased mind*, and that at the time he committed the act he was not *conscious of right and wrong*." Such is the view which the law takes regarding the plea of insanity, and on which it generally proceeds. We say generally, because exceptions have already occurred; for instance, the case of "Regina *v.* Frost," in which Mr. Justice Williams, in his charge to the jury, told them, "it was not merely for them to consider whether he (the prisoner) knew right from wrong, but whether he was, at the time he committed the offence, deranged or not;" thus making the mental derangement itself the ground for acquittal. The capacity of distinguishing between right and wrong being, without doubt, the received test for deciding the legal liability of an accused party, it is well to inquire into its signification. What then does the law, or what do the judges mean, by the terms right and wrong? Do they mean moral and immoral, or lawful and unlawful? Do they refer to divine or human laws? The language employed by some of the most eminent ornaments of the bench, in different cases, clearly shows that they spoke with reference to the moral law,* whilst the terms employed by the fifteen judges, in the opinion above quoted from, seem to make the law itself the standard of wrong. They said—"Every person was supposed to know what *the law* was, and *therefore* nothing could justify a wrong act, except it was clearly proved that the party did not know right from wrong." This difference of language leaves the point undecided, for there is an obvious distinction between moral wrong and legal culpability; many acts are wicked and immoral which are not illegal; while others may be illegal, though not essentially immoral.

* See the Charge of Lord Mansfield, in Rex *v.* Bellingham; and that of Lord Lyndhurst, in Rex *v.* Offord, in both of which they speak of "crime against the laws of *God and Nature*."

We will not, however, insist on this difference; for as human laws are supposed to be based in principle on the divine law—the moral law being the soul or animus of all human legislation—the greater principle necessarily comprises the lesser. Still we think it desirable that a science which sets so great a value on verbal exactitude, and attaches so much importance to technicalities, should be precise in the meaning of the language it employs, and should make it understood, that when it speaks of an act being right or wrong, it means lawful or unlawful. The legal exemption of a party from the liability to be punished for an illegal act, consists, then, of two conditions: first—an insane state of mind; second—unconsciousness of the illegality of the act. We have already shown that the sole legal test for the first of these conditions, the insanity, is capable of merely partial application. Has the law any more comprehensive or exact test of consciousness? What kind of proof does it require to show that the accused party, at the time of committing the offence, was conscious of acting against the law?

The lawyer would perhaps answer, that the law does not ask for positive proof of such consciousness, but supposes every person to know what is legal or illegal, until proof to the contrary is adduced. It is the state of unconsciousness, not that of consciousness, which it requires should be proved. But unconsciousness, being a negative condition, cannot be proved directly. By what method then, does the law investigate this condition? For the sake of clearness, we propose to conduct our argument on this head in the interrogative form. We imagine ourselves conversing with one learned in this branch of the law, and we ask him:

If an accused party is proved to have laboured under a delusion, consequently to have been legally insane, does that excuse his crime?—Certainly not; his delusion may not in any way have influenced his crime.

But in the present instance it is shown that it did, for the illegal act was committed under the immediate impulse of the delusion; does that excuse him?—Far from it; you must now prove that, at the time he committed the act, he was not conscious of its illegality.

But how is that want of consciousness to be proved?—By reference to the circumstances attendant upon the act; to the known character, and previous history of the accused; to his behaviour immediately before the committal of the act; to the violence and inconsequence of the deed itself; to the want of all ordinary motives, such as revenge, anger, or cupidity; to his conduct after the act was committed; to the absence of any precautions to elude detection or secure safety by flight.

Very good, that will do.—Nevertheless, it does not affect the immediate question proposed, nor prove the absence of consciousness; it proves the want of many motives and sentiments common to humanity, but it does not prove any want of knowledge of the law. These circumstances are evidence as to certain conditions or states of mind, similar in character to consciousness, but not identical; for the consciousness of legal right and wrong being a definite state of mind, it follows that your accepted proof amounts only to an attempt to show the non-existence of a certain state of mind, by reference to the absence or presence of certain other states of mind, essentially different in nature, and with which it has not any immediate or necessary connexion. For having no method for *directly ascertaining* the state of a party's mind on a certain occasion with reference to a stated act, you seek to elucidate it, by inquiry into *other contemporary states of mind as evidenced by other acts;* so that your legal investigation becomes a psychological problem, and the question is brought into the domains of a special science, which is not that of the law. But the evidence or absence of design, of all ordinary motives, of pity, of remorse, of caution, of the sentiment of self-preservation, from which you deduce your conviction of the simultaneous want of legal consciousness, though no evidence *pro hac vice*, is strong evidence of an impaired condition of the mental faculties generally, and goes far to demonstrate *radical* unsoundness of mind. But this is merely coming round again to the point already established—viz. the insanity.—Yes; but it proves more than the mere existence of insanity; it demonstrates an amount of unsoundness of mind sufficient to exempt the accused party from legal responsibility.

Then you admit that it is the relative amount of insanity, not the consciousness of right and wrong, which is the real legal test of the liability of an accused party admitted to be insane?—Perhaps it is so: you are aware that the law acknowledges two specific states of insanity—partial insanity and total insanity.

It does; but does it distinguish those conditions? does it define the limit at which partial insanity passes into total?—Certainly. It was Lord Hale who first laid it down that insanity may be general, or it may be partial. "There is," says he, "a partial insanity of mind, and there is a total insanity;" and the former he says is expressed by the phrase "*quoad hoc vel illud insanire*" (1 P. C. c. 4, s. 2). Sir John Nicholl (and Hale does not differ) speaks of "partial insanity as only that which is occasionally called forth, and not that which only exists occasionally" (Brougham).

Just so: what the lawyers call partial insanity, medical men call "monomania;" meaning thereby a state of mind in which a person is uniformly irrational upon some particular subject, or group of subjects, but at the same time perfectly rational on all other matters: how does monomania or, as you term it, "partial insanity," excuse an offence?—

I have already stated that it does not, unless accompanied by a state of unconsciousness of right and wrong.

But I have demonstrated the impossibility of directly proving that particular state of mind, and shown that it can merely be made presumptive by establishing such an amount of disorder of the mind as does not accord with the legal definition of partial insanity, but renders it general insanity. Now, does general insanity excuse an offence?—Assuredly.

It seems then, touching this plea of insanity, that although the law, in theory, requires proof, first of the insanity, and secondly, that the insanity is of such a character as to destroy the capacity of understanding the legal relations of an act; yet in practice, the decision turns upon this point—is the insanity partial or general?—If shown to be only partial, it does not exonerate the accused; if general, it does.

In concluding this interrogation we ask, Who then are the fittest persons to investigate a case of alleged insanity? Common sense would select those who are engaged in the care and treatment of the insane. The law, however, holds a different opinion—it disregards medical evidence, and leaves the judgment of the matter to men, many of whom never saw a case of insanity in their lives. It certainly allows medical witnesses to be examined (yet will not always hear them), and permits them to give a general scientific opinion on some supposititious case constructed to resemble that of the prisoner at the bar, but it silences them when they attempt to give a direct opinion on the case before the court.

We fully understand the grounds on which the legal objection to medical evidence, in these cases, is raised. An opinion as to the existing state of a prisoner's mind at the time of trial, founded on personal examination, is good evidence; but an opinion founded wholly on the observation of others as to the probable state of a prisoner's mind at some antecedent period—viz., at the time when the crime was committed, is not legal evidence, and, notwithstanding it has been sometimes admitted, is commonly rejected. Unfortunately it rarely happens that a medical man has an opportunity of testifying as to the state of mind of an accused party at the precise time when the crime was committed, for he is not usually required to make his examination until the attorney for the defence is getting up his case, that is, shortly before the trial, and generally some months after the date of the offence. In this interval a great change may have taken place in the prisoner's mind. On the one hand, a person decidedly insane at the time of committing an offence may have perfectly recovered his reason; whilst another, sane when the crime was committed, may have become insane from remorse, imprisonment, and the anxiety of mind arising from his position.

Since the law requires *positive evidence* that the accused was of diseased mind *at the time* he committed the offence, it follows that unless the medical witness can testify this from *personal observation made at the time,* his testimony has no legal value. The function of the witness is solely to relate facts; a conclusion founded on circumstances related by other witnesses, *i. e.* upon *hearsay,* not being fact, is not legal evidence, but merely opinion, which the court has a discretionary power to admit or reject. We think it excusable for us to have directed special attention to this point, on account of the false position in which medical witnesses are frequently placed by disregarding it. It is well known that our judges are kind-hearted and conscientious men, willing, on every possible occasion, to temper justice with mercy; but they have their professional prejudices, and when they refuse to listen to medical witnesses on the plea of insanity, they are actuated, not by a wish to stifle the voice of humanity, but by a predilection for established legal forms, and a jealousy of any infringement or innovation of the law of evidence.

However, now that a radical reform of the law is in agitation, and the profane hand of parliament has already meddled with the venerable structure of the law of evidence, we hope that some provision will be made to admit of medical opinions being received as evidence, even when formed solely on the sworn testimony of other witnesses, without personal knowledge of the facts. This is one of the points to which the committee appointed by the "Association of Medical Officers of Hospitals for the Insane" for suggesting amendments of the law relating to lunacy should direct its attention. We cannot see what danger or inconvenience could result from the proposed arrangement; the evidence of the medical witness would not decide the case; his opinion would go to the jury with the rest of the testimony, after having been analyzed and sifted by the judge, and the jury would estimate its value in accordance with their oath. Whatever might be the practical results of the alteration, one of its agreeable effects would be, that it would enable the medical practitioner conversant with insanity to appear in the witness-box, without having to dread the interruption of the court, and the gentle admonition, "not to take upon himself the functions of the judge and jury." In the meantime whilst waiting for this desirable change, we respectfully suggest to our judges, that they should exercise the power they possess, of staying counsel in putting an improper leading question, instead of reprimanding the medical witness for answering it.

Moreover, there is frequently a disposition on the part of the Bench, not merely to reject medical evidence on technical grounds, but also to depreciate the scientific value of such evidence. One judge (Denman) says that "doctors are in the habit of making theories," and tells a medical witness that "his opinion had been very rashly formed." Another judge (Campbell) tells the jury, that the medical witnesses in a case "might just as well have stayed at home, and attended to their patients." Another judge (Alderson) says he will not allow any medical witness to usurp the functions both of "judge and jury;" and Lord Chancellor Truro is reported to have said, "his experience taught him there were very few cases of insanity in which any good came from the examination of medical witnesses. Their evidence sometimes adorned a case, and gave rise to very agreeable and interesting scientific discussions; but, after all, it had little or no weight with a jury."

From these expressions it would seem that medical witnesses are looked upon as *intruders* in the case, and supposed to be actuated by an inclination to busy themselves about matters with which they have no real concern. We indignantly repel an imputation so uncalled for and unjust that we are at a loss clearly to account for it. We suppose it proceeds from a supposition that every man, of sound mind himself, has an intuitive perception of the characteristics of sound mind in others, and therefore is a competent judge upon all questions concerning the integrity of the mental faculties. But is it so? is this supposition a legal fiction or a fact? For our own part we do not consider the operations of the human mind so self-evident that a critical knowledge of them can be obtained without careful study and reflection. In the class of citizens from whom juries are usually selected how few are learned in the science of the mind, or capable of analyzing the most simple mental process. We shall perhaps be told that juries have not to deal with nice metaphysical distinctions, but plain common facts; nevertheless, we think we have proved that, whatever the law on the plea of insanity may be in theory, in practice the question resolves itself into a psychological inquiry, which the jury has to decide. And, moreover, that whilst it imposes upon men unaccustomed to investigate the natural and healthy operations of the human mind the task of judging of its aberrations, it frequently seeks to deprive them of the assistance which the science of men practically conversant with the subject may be capable of affording them. Furthermore, even when the jury has decided the simple question of the insanity, it has accomplished only half its duty; the more important inquiry, the inquiry by which the law determines the culpability of the accused—viz., the amount of the insanity, remains to be undertaken.

This inquiry is altogether distinct from the preceding. The law may be of opinion that insanity *per se* is so evident, so palpable and easy of detection, as to make the recognition of it perfectly sure and simple to every sane man possessed of common understanding; consequently, that it is too obvious a matter to require scientific aid for its discovery; but surely it does not and cannot assume that every man is by nature and intuitively a competent judge of the character, amount, and quality of the first case of insanity presented to his notice? If such be the legal assumption, why does the law (8 & 9 Victoria, cap. 100, sect. 45) intrust the responsibility of advising the confinement of insane persons *exclusively* to medical men? Does it not seem, by this very regulation, to restrict the legal competency of determining the relative amount of a person's insanity solely to members of the medical profession? It seems so to us, and we think it acts wisely in doing so. For admitting that every sane man is competent to judge of the general sanity of a party, it by no means follows that he is a competent judge of insanity. Hence the necessity of allowing the jury engaged in deciding upon a plea of insanity the assistance of professional men presumably conversant with the subject—viz., those specially occupied with the care and treatment of the insane. We will not here repeat the scientific reasons why medical psychologists should be consulted in every investigation concerning the state of a person's mind; they have frequently been discussed in the pages of this journal, and some are ably treated of in Dr. Wood's pamphlet before us. We have chosen, on the present occasion, to consider the subject in a legal point of view, and we think we have shown—1stly, that the law has no universally applicable test of insanity; 2ndly, that it has no practicable test by which it can determine the responsibility of insane persons; 3rdly, that a judicial investigation of the plea of insanity is in fact a psychological inquiry, and, as such, requires the aid of persons specially conversant with the subject. Moreover, as the law of evidence does not at present admit of this, we have suggested such a change as would meet the exigencies of the case.

In the foregoing argument it is assumed that the plea of insanity is based on sound presumptive grounds. Unfortunately, such is not always the case, for it is sometimes adopted as a sort of "forlorn hope," constructed on very slight foundations, and supported by most inconclusive evidence. With such cases we earnestly entreat our professional brethren to have nothing whatever to do; unless the medical witness is prepared (if allowed by the court) to express a very decided opinion, and can support that opinion by circumstantial reasons, it were much better for him not to appear in the case. It is not sufficient that he shall himself clearly comprehend the data upon which his own opinion is formed, but he must be able to expound those data, so as to render their nature and import comprehensible to every man of common understanding. He must carefully avoid too curious metaphysical speculations, and guard against overstraining the evidence, and against allowing his judgment to be warped by a professional bias; above all, he should give his evidence in the most clear and simple language at his command, and in a firm and dignified manner.

We find many judicious remarks on some of the points discussed in the preceding pages, in Dr. Wood's pamphlet. He says:—

"Medical witnesses have some fair ground of complaint, not only that their evidence is not received with the consideration to which it is entitled, but that they are sometimes forbidden to express the opinion which is the real object of their appearance in court. It is not contended that a physician is necessarily more competent to decide on the insanity of an individual than a lawyer, or any other intelligent person who has paid the same attention to the subject; it is not for a moment supposed that others, and especially those who have devoted themselves to the severer study of the law, are not quite as capable, with the same experience, of arriving at a just conclusion; but it may be fairly argued, that one who has made the ever-varying forms of mental disturbance his constant study must be more competent to weigh the evidence for and against the reality of the alleged insanity, than one who has never had such opportunities, and has been, therefore, without the means of learning practically in what insanity consists. I apprehend that the proper duty of the medical witness is to assist the court with his experience and advice to arrive at a just decision in the particular case; it is not to say what symptoms are most frequently observed in insane persons, or to deliver abstract opinions on the nature of insanity generally, but to deal with the individual case; to give the court reasons why he adopts the conclusions at which he has arrived; to state, in fact, what are the particular circumstances which have led him to form his opinion of the case; in other words, to say why he thinks the accused sane or insane, as the case may be, and then leave the jury to determine whether the reasons are sufficiently satisfactory to induce them to adopt his opinion. If this course were adopted, it could not be said that the medical witness usurped the province of the jury; he did, in fact, but assist in guiding them to a right decision; and, indeed, without this assistance, which could only be rendered by the medical witness, they would be left to form their own conclusions, with the great danger to the cause of humanity and justice of thinking more of the nature of the offence than of the probable irresponsibility of the accused. I would not be misunderstood as urging that the mere dictum of a physician, whatever his reputation or opportunities of forming an opinion, should be taken as conclusive evidence, unless he could give good grounds for the conclusions at which he had arrived; but surely he should be heard when he endeavours to explain those grounds, for it remains for the judge to point out to the jury the weak points of his evidence, the fallacies of his reasoning, and the unsoundness of his arguments. It seems, then, to be an error in the practice of our criminal courts, to restrict the medical witness from a free discussion of the merits of the case." (pp. 22, 23, 24.)

We entirely coincide with the opinions and sentiments expressed in the foregoing quotation, but not with that contained in the following passage. "I conceive that the humanity of medical witnesses has induced them to be content with too little direct evidence of insanity." (p. 20.)

We cannot conceive that any conscientious physician would allow his feelings to overcome so far the solemn obligation of his oath. He has no concern with the legal criminality of the act, nor with the kind or degree of punishment incurred by it; he has to testify to the state of mind of the accused—to that and to no issue beside; and if he, from any misplaced commiseration, or influenced by matters beyond the functions of his office, should pronounce an accused party to be insane, upon a weak, partial, and imperfect conviction of the insanity, he would be beyond question morally guilty of perjury of the most dangerous kind. Still, whilst unwilling to believe that a medical witness could so far forget the nature of his oath as to swear a mere suspicion or presumption for a certitude, we admit that he may sometimes commit an error of judgment, owing to the inadequate opportunities afforded him for investigating the prisoner's state of mind. As Dr. Bucknill observes—

"Generally, the physician giving evidence can at most say, that he has paid two or three visits to the accused, and conversed with him in his cell in prison; sometimes he can only say that he has observed the demeanour of the prisoner in court, and has heard the evidence of other witnesses, from which he forms his opinion. In cases of concealed delusions, or of disease affecting the propensities, no medical man ought to give an opinion on such shallow grounds. I am not ashamed to acknowledge, that I have often observed patients daily for several weeks, without being able to detect existing delusion. The plan adopted in France, of sending a supposed lunatic to an asylum, for observation before trial, meets this difficulty." (p. 36.)

But whether the opportunities for observation have been few or ample, we repeat our advice, that unless the medical man "be fully persuaded in his own mind" of the accused's insanity, he should abstain from giving evidence in the case.

Our readers are aware that the question of establishing a central asylum, appropriate to the reception of "Criminal Lunatics," in England and Wales, similar in character to the asylum at Dundrum, near Dublin, for all Ireland, has been warmly agitated during the past twelvemonth. The suggestion of the necessity for some such establishment came originally, we believe, from the Commissioners in Lunacy, who, in their fourth annual report (1849), directed the Lord Chancellor's attention to the subject.

In our last number we gave an abstract of the discussion at the late meeting of the Association of Medical Officers of Hospitals for the Insane, together with some letters and remarks from the public press upon the same matter. Since then a letter, written by Dr. Boyd, has appeared in one of the daily papers; and now we have Drs. Wood and Bucknill treating the question with equal ability and vigour, but differing materially in their respective views. Dr. Wood proposes that the central asylum shall be called the "State Asylum," and that it shall be restricted to the reception of all criminal offenders, of every station and degree, exempted from the penalties of the law on the ground of insanity. Dr. Wood proposes that this institution shall have the character of a general, and not a pauper asylum, so as to afford superior accommodation for such of its inmates as can afford to pay for it. Dr. Bucknill, on the contrary, thinks "that a distinct institution is not necessary for the treatment of *all lunatics* in detention under warrant from the Crown or Secretary of State, generally called criminal lunatics;" but "that such an institution is desirable for the detention and treatment of lunatics of *criminal disposition*, many of whom are not criminal lunatics." (p. 7.) Dr. Wood objects to placing criminals, who have become insane after conviction, on the same footing with the other inmates of the "State Asylum;" but Dr. Bucknill would make no distinction. Moreover, Dr. Bucknill would treat all "state lunatics" as paupers, and not make any difference on account of the lunatic's means and previous position in society. On all of these points we concur with Dr. Wood. We think the name "State Lunatic Asylum" very appropriate, and the title "State Lunatic Patients"* well adapted to designate the inmates of such an establishment. We would completely separate criminals who have become insane after conviction from those who have become criminals from insanity; and we think the former would be properly styled "Insane Convicts." In common with Dr. Wood, we strongly object to the term "criminal lunatic;" a criminal who becomes insane after conviction is an "insane convict,"—*i.e.*, a convict affected with insanity,—but an insane person cannot become criminal after his insanity is recognised.

Dr. Wood justly remarks—"The name criminal lunatic is sufficiently inappropriate to any class, for it conveys a contradiction of terms, inasmuch as the law holds that the lunatic is not criminal, and that he should always be acquitted of whatever crime he may commit as a lunatic." (p. 52.) We also agree with Dr. Wood in thinking that a difference of accommodation should be allowed to "state lunatic patients," whose friends may be willing to defray the additional expense. There is no reason why a "state lunatic patient" should be treated differently from any other lunatic of his own rank and station

* Dr. Wood proposes to call them merely "State Patients," having an objection to the word lunatic, as being founded on a vulgar error. The word is, however, so established in the language, that we see no reason to discard it.

in society. Why should a gentleman who, impelled by an insane impulse, has violated the law, be degraded from his sphere of life, and be constrained to associate with persons whose habits and language are uncongenial, and perhaps offensive, to him? Is his insanity, itself a sufficiently grievous affliction, a cause why he should be debarred from every alleviation of his unhappy state, and be exposed to daily outrage of his feelings? Certainly not: the lunatic who commits an illegal act does not thereby become a criminal, and should not be regarded as a felon, but should be taken care of, and treated with the gentleness and humanity which the insane ought invariably to receive, and allowed every reasonable indulgence consistent with his safe keeping. Dr. Bucknill has some remarks on this head which we cannot suffer to pass unchallenged. He considers that the "principles of justice" would be violated if any distinction were made in the treatment of criminal lunatics, forgetting that lunatics are irresponsible agents, and therefore not answerable to justice. Moreover, he says, "It would stamp poverty with inferiority, and deny to gentility the possibility of ever being subject to ruffianly propensities. If patients are not criminal, why confine them in a State asylum?—if they are criminal, why keep up unjust social distinctions?" (p. 5.) How came it not to strike Dr. Bucknill that patients are confined in an asylum, not because they are *criminal*, but because they are *insane*. If a person is tried for a criminal act, and acquitted on the ground of insanity, he stands absolved of all criminality, and is sent to a lunatic asylum to be taken care of as a patient, not as a criminal. We are the more surprised that Dr. Bucknill should have fallen into this error, since we find him, a few pages further on, correctly stating the real bearing of the case in these words,—"When a prisoner is found, on trial, not guilty on the plea of insanity, he is legally as free from criminality as if it had been proved that the offence charged had never been committed, or had been committed by some other person; under which supposition *no punishment* ought to be inflicted on him, beyond *the treatment* necessary for the safety of the public, and the cure or alleviation of his disease." (p. 19.) Here, again, we differ with Dr. Bucknill. We hold that on no occasion, and under no circumstances, ought *punishment* to be inflicted upon the person of one whose insanity has been established by the verdict of a jury, and we strongly reprobate the use of the word punishment in connexion with the treatment of the insane, and protest against making lunatic asylums penal establishments. The insane are, or should be, subject to *discipline;* they may have habits and propensities which require *correction;* but they cannot, from the very nature of their malady, become liable to *punishment.* The word punishment (from the Latin *punio,* and the Greek πεινη, pain,) communicates the idea of suffering designedly inflicted, and cannot, therefore, refer to any part of the treatment of insanity. Nor is our objection at all modified by Dr. Bucknill's explanation of the meaning he attaches to the term; "I admit," he says, "that if lunatics can distinguish right from wrong they are liable to punishment, as understood in the philosophic sense of the term; correction modified to their condition, and containing no spirit of revenge, even in the most subdued and unrecognised form" (p. 31); for only in the preceding page he says—"The feeling of right and wrong, otherwise called conscience, is an innate principle of the human mind; and probably no state of disease, short of utter loss of mind, altogether destroys it" (p. 30); according to which it would seem that Dr. Bucknill considers every lunatic, not wholly demented, a fit subject for punishment. We certainly cannot acquiesce in this extreme opinion; a lunatic is to be prevented from indulging in evil habits and propensities, so far as is possible, by constant supervision, by moral influence, by seclusion, and even by restraint; but if he cannot be prevented or restrained by these means, he is not to be corrected by the infliction of pain. It was this very idea, that madness may be cured by punishment, which led to half the cruelties of the old method of treatment. Finally, in respect to criminal lunatics, it most assuredly is not the province of the medical psychologist to punish those whom the law professedly exempts from punishment.

Reverting for a moment to the question whether there should be any difference in the accommodation of patients in the "State Lunatic Asylum," we think that if such an arrangement should be considered objectionable, permission might be given to the friends of state lunatic patients of the middle or upper ranks in life to place them in private asylums. Of course the proprietor of the asylum would have to guarantee the safe custody of the patient under such penalties as the law might impose. In fact, under any circumstance, the friends of an insane person, able and willing to pay for his support, should have the option of placing him where they might think best.

One of the reasons adduced by those who advocate a separate asylum for the so-called "criminal lunatics," is that mentioned in the fifth report (1850), of the Commissioners on Lunacy: they say "it has been frequently brought under our notice that the friends and relatives of patients, and also the patients themselves, when conscious of their being associated with criminal lunatics, have considered such association as a great and unnecessary aggravation of their calamity." (Report, p. 17.) Dr. Bucknill has directed his attention to this point, and we are pleased to find that his conclusions do not tally with those of the commissioner. We are of opinion, that even in those cases in which so uncharitable a feeling can be shown to exist, it may rightly be attributed to a prejudice derived from the cruel inconsistency of the law, which brands the insane offender with the name of criminal, and practically treats him as one, after formally absolving him of his crime. The following is Dr. Bucknill's testimony. To the question "Whether the dislike to the society of criminal lunatics ascribed to other patients and their friends, exists generally, or only in those cases which have been brought under the notice of the commissioners?" he answers:

"My own experience is in direct opposition to that which has been recorded by several of my professional brethren. I have carefully watched to detect any repugnance or unfriendly feeling among the inmates of this establishment (the Devon County Asylum) towards their fellow patients who were known to have committed offences against the laws, and have not only failed to do so, but have heard expressions of sympathy and pity. The case of No. 12 excited much interest and discussion in the wards, and the poor woman, who is now at large, has reason to contrast most favourably the treatment she received from her fellow patients with that she has met with from her sane neighbours since her discharge. The insane, while they are unable to appreciate their own mental condition, are keen observers with regard to others, and they excuse an offence, the evident result of insanity, as it is excused by law and the common consent of mankind." (p. 17.)

The case above referred to is related by Dr. Bucknill in an appendix.

"An artizan's wife; married below her position in life, and was exposed to much family discord and very straitened circumstances. Her friends and elder children observed that she was becoming strange; and one morning, having been *without food* nearly the whole day, she induced her three younger children to go out with her for a walk, and threw them into the canal; two were drowned. She remained in gaol for some time, but was not put on her trial, because she was too ill to plead to the indictment. On removal to the asylum, she was in a very precarious state from bodily illness, had an idea that she was brought from a nobleman's house, and was quite unconscious of the destruction of her children. She soon recovered health of body, and appeared to be of sane mind, although her memory for recent occurrences was a blank. At the ensuing assizes an attempt was made to put her on her trial, but as no notice had been given that such a step would be taken, she had been allowed to remain in ignorance of the death of her children and of her own position. When this was communicated to her, in order that she might plead, it produced so much mental agony that the court determined not to proceed with her case. At the end of six months she was again put on her trial, and acquitted because no evidence was offered. In the asylum she was industrious, and although unamiable, was harmless and quiet. Since her liberation she has been engaged in many quarrels with her neighbours, who have taunted her with her misfortunes, and she has even been obliged to seek the protection of the bench of magistrates on this account." (p. 49.)

There can be no doubt about the state of mind of this poor creature when she committed the murder; she was evidently labouring under delirium caused by starvation.

One of the grounds on which Dr. Bucknill advocates the construction of a distinct asylum for state lunatics is the defective and unsatisfactory arrangement for their accommodation at Bethlem Hospital. He feelingly remarks on the bare and desolate aspect of the wards appropriated to their use in that institution (than which anything more prison-like can hardly be conceived), and shows, not only on humane, but also on economic grounds, that some new arrangement is desirable.

"In the past month the number of male patients in this place was 92, of whom 36 were convicts; the number of females was 19, of whom 4 were convicts. On account of these 111 criminal lunatics, the hospital is repaid by the government about 3500*l.* per annum, being at the rate of 31*l.* 10*s.* 7*d.* each per annum. This sum is made up by the wages of seven male and two female attendants, with an allowance of 35*l.* per annum to each attendant for maintenance and clothing, 25*l.* per annum for each patient, and 200*l.* per annum for salaries to medical and other officers. During the last year the actual cost for maintenance and treatment of pauper lunatics in the county asylums for Middlesex and Surrey, was 7*s.* 6*d.* a week, or 19*l.* 10*s.* each, which, deducted from 31*l.* 10*s.* 7*d.*, would leave 12*l.* 0*s.* 7*d.* as the actual rent for the use of the building paid for each criminal lunatic in Bethlem. Few asylums have cost so much as 200*l.* a bed, and this, when building was much dearer than at present. As Government can borrow money at three per cent., and keep a building in repair for one-half per cent. more, state lunatics could, even on this estimate, be lodged in a new asylum, with the best accommodation, at an annual rental of 7*l.* each, while 12*l.* is at the present time given for the worst. I am not aware that anything can be said in favour of maintaining the arrangement existing between government and the authorities of Bethlem Hospital, and when this is concluded, a new establishment must be provided." (pp. 24, 25.)

We now pass to those important portions of the pamphlets before us, in which the respective authors treat of the comparative responsibility of the insane. We have explained above that the law recognises two species of insanity, partial insanity and total insanity; of which, total insanity alone exempts from legal responsibility, partial insanity being no excuse, nor even a plea in mitigation of punishment. Between these two conditions, entire responsibility and complete exemption, the law admits no intermediate state.

We quote at length Dr. Wood's remark on this deficiency, and his proposal for remedying it—

"Unquestionably the great defect of our criminal code is this, that different degrees of guilt are not recognised, and the magnitude of the defect is particularly felt in those cases where the plea of insanity is urged as an excuse for crime. We either allow this to the fullest extent, and nominally acquit the accused, or we reject it altogether, and impose the full penalty. * * * If we were to recognise as a principle the different degrees of insanity, and, as our neighbours do, the different degrees of moral guilt, the difficulty would be at once removed; we should not be left to the alternative of the full punishment or the absolute acquittal; the medical evidence would be received with more consideration than it at present obtains, for it would not be limited strictly to the question of sanity or insanity, but to the degree which had been really manifested by the accused; a less amount of responsibility would devolve upon the medical witness than at present, and the court could therefore afford to receive his evidence with less jealousy and caution.

"According to the present system, where there is no middle course between the two extremes, the fate of the accused is really in the hands of the jury, as it ought to be. But the judges have seen and have endeavoured to remedy the defect by restraining the medical witness from entering fully into his views of the matter, lest he should prejudge the case too much in the minds of the jury; the effect of this must be to leave the jury without that information which it is impossible for them to get from any other source. We can scarcely wonder that any single individual should shrink from the appalling responsibility of declaring an alleged lunatic sane, when he knows that such an opinion was a death-warrant to the accused; nor can we wonder that the judges should endeavour to impose this responsibility on the jury.

"If our law allowed the jury to declare a verdict of 'GUILTY, WITH EXTENUATING CIRCUMSTANCES,' in the first or second degree, all those doubtful cases which now attest the imperfection of our system would be properly dealt with; a degree of punishment or restriction would be imposed in exact proportion to the degree of moral guilt, modified by the mental condition of the accused, and there would then be no objection to hearing all that a medical witness could say for the defence, whilst he would have less hesitation in declaring the criminal sane, if the circumstances of the case tended to that conclusion; he would be relieved of all responsibility as to the fate of the accused, if there yet remained with the jury the power of averting the extreme sentence, by appending to their verdict of guilty that there were extenuating circumstances." (pp. 45, 46, 47.)

We will next quote Dr. Bucknill's observations on the same subject:—

"In cases of murder our law permits juries to bring in a verdict of guilty or not guilty only; and if the former, no course is open to the judge except that of passing sentence of death. Until some middle way is devised by which offenders, neither altogether innocent nor altogether guilty, can have their proper meed of correction, juries, in cases of murder, will continue to find verdicts of not guilty on the false plea of insanity. As the power to attemper justice with mercy is accorded to neither judge nor jury, the latter seize the only opportunity to ensure mercy, and leave justice to take care of itself. In some other countries where trial by jury is established, some greater latitude is permitted in recording a verdict than in our own. In France a verdict of guilty, with extenuating circumstances, and in the Channel Islands, a verdict of 'more guilty than innocent' are permitted. I would not advocate any extension of power to juries, but I do think that discretion in awarding punishment for murder ought to be vested in the hands of our judges. They possess it for other crimes—thus, for manslaughter, they sentence one criminal to one month's imprisonment and another to transportation for life, according to the character of the particular offence, and the existence or not of extenuating circumstances. If our judicial authorities possessed this discretionary power, a state asylum for the detention and correction of lunatics of criminal disposition would provide the means for the exercise of it when murder was extenuated by partial insanity. At present, society may either be shocked by the execution of a madman, or it may be endangered by his acquittal. It is true that when a man is acquitted of a murder on the plea of insanity he does not, except in rare instances, obtain his liberty; the law steps in and detains him during the Queen's pleasure, which usually means for the term of his natural life. When the plea of insanity has been false, this detention can only be considered as punishment under the guise of a legal fiction. Surely it would be more wise to make arrangements by which suitable correction could be ensured in an honest straightforward manner, than to force juries to acquit, and then to say to the man, late a prisoner, and now a patient, 'You shall not escape; sane or insane the law shall have it out of you; imprisonment for life is your lot, whatever may be the state of your mind.'" (pp. 33, 34, 35.)

These extracts show that the authors, whilst equally condemning the present inflexibility of the law with respect to the plea of insanity, are not quite agreed as to the method of amending it. Dr. Wood would authorize the jury to extenuate the crime. Dr. Bucknill would allow the judge to mitigate the punishment. The practical result of the two proposals would be identical, but the form of the latter is more in harmony with the structure of the legal procedure in criminal inquiries. In our Constitution, the Sovereign is the sole fountain of mercy and clemency, and the only authority in the state possessed of power to arrest or divert the course of justice; consequently, the judge, being the representative of the sovereign, is rightly allowed to exercise, within certain limits, a personal discretion in fixing the amount of a culprit's punishment. On this ground we are opposed to the change advised by Dr. Wood. Moreover, we have paid some attention to the administration of criminal justice in France, and we are convinced that the rule which permits the jury to return a verdict of "guilty with extenuating circumstances," counteracts the main purpose of criminal justice, which is the suppression of offences by the penal correction of offenders. Since the publication of Beccaria's celebrated treatise, legists are almost unanimously agreed that the efficiency of penal codes in the prevention of crime, depends not so much on the relative severity as on the certainty of punishment. On this account the French process is strongly objectionable, for the verdict of "guilty with extenuating circumstances" is destructive of all certainty and uniformity in the administration of the criminal law. It is frequently a difficult matter to guess at the probable motive which induces a French jury to discover "extenuating circumstances." In a terrible case, that of the friar Léotaud, tried at Toulouse, in 1848, for the murder of a young girl after violating her, a verdict of "guilty with extenuating circumstances" was returned, although the only plea for extenuation that could possibly arise out of the case was the vow of chastity of the accused. And in the case of Madame Laffarge "the extenuating circumstances" were a disagreeable husband and an agreeable lover.

But whilst deprecating the introduction of a new principle in the administration of criminal justice in this country, we see no objection to the further extension of an old established form. The law of England, although it restricts the exercise of mercy to the judge, yet allows the jury to supplicate that mercy in the form of a recommendation, which, if based on circumstantial grounds, always receives due consideration from the court, and often materially influences the sentence. The ground on which the recommendation to mercy is preferred is not always stated by the jury, but sometimes it is, and sometimes it is demanded by the judge. Now, we propose to make it compulsory for the jury always to assign the motives of a recommendation to mercy. If such a rule obtained, what more valid motive could a jury adduce than that of a doubt respecting the perfect sanity of the prisoner? Cases are continually occurring in which the evidence, though insufficient to warrant an acquittal on the ground of insanity, is conclusive as to the existence of such an amount of mental derangement as should serve to extenuate the prisoner's guilt and mitigate his punishment.

When the unsoundness of mind is found to amount to what the law terms total insanity we would retain the present form of verdict; but in those cases of doubtful character, in which proof of some previous attack of insanity, or habitual eccentricity, or recent change of sentiment and conduct, not amounting to evidence of positive lunacy, and having no immediate connexion with the crime, yet sufficient to suggest the suspicion of so much mental unsoundness as would tend to impair the will, obscure the judgment, and preclude a full, clear, and just apprehension of the criminality of an offence; in such cases we would authorize the jury to return a verdict of guilty, with a recommendation to mercy on the ground of presumable insanity. In this manner the law would be provided with a suitable finding for each of the two degrees of mental derangement which it recognises :—"*Guilty; but recommended to mercy on the ground of presumable insanity,*" would be the appropriate verdict for all cases of that kind of insanity which the law calls partial, and "*Acquitted on the ground of insanity,*" would, as heretofore, be the verdict in all cases of total or general insanity, The judge would have to decide on the propriety of the recommendation to mercy, and would regulate his sentence accordingly: if he agreed with the jury, he would probably defer sentence so as to afford time for a further inquiry into the state of the prisoner's mind; if, on the contrary, he differed in opinion with the jury, he would proceed to pass sentence immediately.

We have never advocated the doctrine that simple aberration of mind, irrespective of its quality or degree, is a sufficient reason for entire exemption from legal responsibility, but we have always considered it a valid reason for a mitigation of punishment. In our remarks on the trial of Robert Pate, we said*—"In every criminal case where the question of responsibility arises in the course of judicial inquiry, if it be possible to establish any degree of positive insanity, it should always be received as a valid plea for a considerable *mitigation of punishment,* and as a *primâ facie* evidence in favour of the prisoner, and in no case where insanity clearly exists (without regard to its nature and account) ought the extreme penalty of the law to be inflicted." In its present state, the law permits no discrimination, for so far as punishment is concerned, it takes no account of the degree of insanity, and recognises no intermediate condition between perfect sanity and total insanity; but the change we have proposed would satisfy the necessities of the case, and render the law more consonant with the enlarged humanity and progressive enlightenment of the age.

We were desirous of discussing other points arising out of the contents of these two pamphlets, but our limits forbid; and we have space only to thank the respective authors for much interesting information and many valuable suggestions.

* Psychological Journal, vol. iii. p. 456.

